# In Vitro Assay Development to Study Pulse Field Ablation Outcome Using Solanum Tuberosum

**DOI:** 10.3390/ijms25168967

**Published:** 2024-08-17

**Authors:** Akshay Narkar, Abouzar Kaboudian, Yasaman Ardeshirpour, Maura Casciola, Tromondae K. Feaster, Ksenia Blinova

**Affiliations:** Center for Devices and Radiological Health, US Food and Drug Administration, Silver Spring, MD 20993, USA

**Keywords:** electroporation, pulsed field ablation, tuber model, potato, lesion depth

## Abstract

Exposing cells to intense and brief electric field pulses can modulate cell permeability, a phenomenon termed electroporation. When applied in medical treatments of diseases like cancer and cardiac arrhythmias, depending on level of cellular destruction, it is also referred to as irreversible electroporation (IRE) or Pulsed Field Ablation (PFA). For ablation device testing, several pulse parameters need to be characterized in a comprehensive manner to assess lesion boundary and efficacy. Overly aggressive voltages and application numbers increase animal burden. The potato tuber is a widely used initial model for the early testing of electroporation. The aim of this study is to characterize and refine bench testing for the ablation outcomes of PFA in this simplistic vegetal model. For in vitro assays, several pulse parameters like voltage, duration, and frequency were modulated to study effects not only on 2D ablation area but also 3D depth and volume. As PFA is a relatively new technology with minimal thermal effects, we also measured temperature changes before, during, and after ablation. Data from experiments were supplemented with in silico modeling to examine E-field distribution. We have estimated the irreversible electroporation threshold in *Solanum Tuberosum* to be at 240 V/cm. This bench testing platform can screen several pulse recipes at early stages of PFA device development in a rapid and high-throughput manner before proceeding to laborious trials for IRE medical devices.

## 1. Introduction

A novel ablation technique called Pulsed Field Ablation (PFA), also known as irreversible electroporation (IRE), that delivers short and intense electric pulses shows promise in fields like cardiology and oncology [1,2,3,4,5,6,7,8,9,10]. Several underlying mechanisms are thought to be triggered when cells are exposed to electric pulses, for example, changes in transmembrane potential, structural rearrangement, and downstream signaling pathways [11,12,13]. Depending on the strength of this external electrical field, the pulse may exert none to significant cellular effects like irreversible death [2,14]. Ablation is multi-faceted, and the outcome depends on several factors including, but not limited to, field distribution, pulse parameters (frequency, voltage, duration, etc.), electrode design, contact force, and tissue architecture. Robust PFA characterization requires a systematic study to delineate upstream pulse signals from downstream ablation effects. Thus, there is a heavy dependance on living cells or tissue and ultimately characterization relies on several animal experiments for safety and efficacy assessment. However, for early discovery, filtration and screening of overly aggressive scenarios including high voltages are critical to minimize harm to animals. In line with principles of 3R (replacement, reduction, and refinement) there is a continuing need for alternate method development to reduce animal burden [15]. Biological cells of plants and animals vary in form and function. Despite differences in the cell wall and plasma membrane, pulsating electric fields can exert similar effects on living cells as exchange occurs between internal cellular material and outside ions and macromolecules. Vegetal cell walls do not prevent the electric field from destabilizing the membrane and eventually causing electroporation [9,16,17]. The end points for evaluating PFA lesions in situ are critical to assessing ablation outcomes. Prior studies have applied a myriad of assays like tissue impedance, fluorescent dyes, histology staining, MRI, and ultrasound with cell lines, animal tissue, plant models, and gel phantoms to investigate ablation outcomes [9,18,19,20,21,22,23,24,25]. While every model has its own pros and cons, certain issues like 3D depth (transmurality) assessment, scalability, technical skillsets, invasive properties, and overly aggressive scenario filtering remain a daunting challenge. In the laboratory, the *Solanum tuberosum* (*ST*), also known as the potato, model is commonly used for electroporation studies, as the lesions can be examined fast and in a noninvasive way [21,26,27]. Numerical models can also be considered as supplementary models to investigate a broad set of pulse parameters due to “black box” generator recipes. This elusive nature also acts as a rate limiting step to quantify and characterize biophysical aspects of PFA.

Here, we report the characterization of the in vitro vegetal model to elucidate ablation outcomes using a comprehensive set of pulse parameters along with numeral modeling. The aim of this study is to demonstrate that this model can be used to create clinically relevant ablation zones by modulating key determinants of PFA including duration, voltage, and frequency. We performed E-field numerical simulations and compared them to lesion boundaries to quantify the lethal electric field threshold (EFT). Refinement and adoption of the vegetal bench assay supplemented with simulations at an early discovery phase can act as screening tool before commencement of laborious animal testing.

## 2. Results

### 2.1. Vegetal Model Characterization to Study Ablation Outcomes

**Figure 1:**
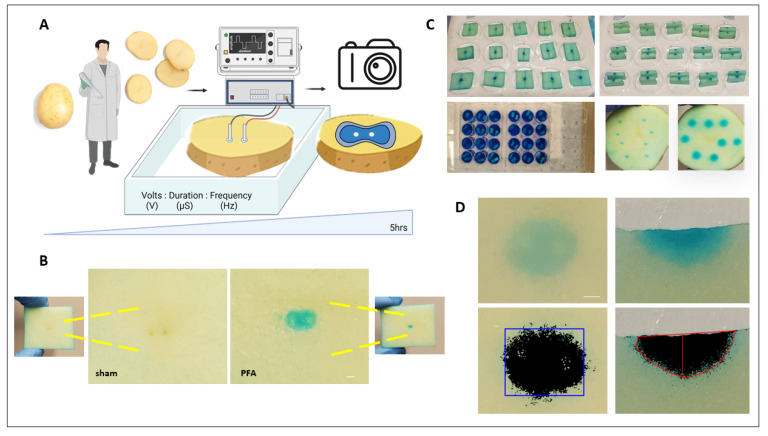
Characterization of in vitro vegetal model to test pulse parameters. (**A**) Workflow schematic using solanum tuberosum slices to test pulse parameters. (**B**) Representative slices with sham control and PFA-treated lesions. Scale bar 1 mm. (**C**) Dye staining of slices in different formats and XY and Z lesions. (**D**) Digitization of slices for image analysis (red and blue lines indicate lesion boundary and dimension) and lesion quantification.

We characterized optimal assay parameters by systematically testing various staining parameters, time points, slice sizes, and incubation methods. An overview of the workflow and assay development parameters is illustrated in Figure 1A. Lesions became visible within a few hours post-treatment. While longer incubation periods were feasible, overnight incubations rendered slices susceptible to increased background oxidation and buckling. For all our experiments, slices were accompanied by sham controls (no PFA) alongside specified treatments. A representative image of sham and PFA-treated slices 3 h. post treatment is shown in Figure 1B. Qualitatively, the needle electrodes produced lesions that resemble oblong peanut shaped to semi-elliptical geometry. We compared traditional discoloration/oxidation (no dye) with the dye staining and found that lesion boundaries were more distinct with the staining protocol (Figure S1A,B). The dye protocol is rapid, and lesions appear in both the XY and Z planes in the first few hours post treatment. The assay can be performed in a semi-high-throughput manner (Figure 1C) as samples are cost effective and readily available; also, each slice can be used to test individual or combinations of pulse parameters, and staining can be performed in multiple well formats. Post staining, digitization of the slice is performed (Figure 1D) using a custom image analysis platform to quantify lesion characteristics like area, length, width, depth, and volume.

### 2.2. Increasing Voltage Increases Lesion Size and Depth

**Figure 2:**
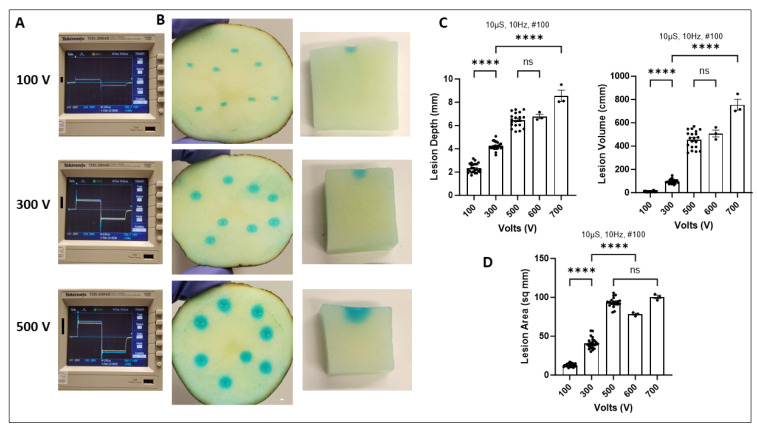
Increasing voltage significantly increases lesion size. (**A**) Oscilloscope recordings with biphasic square waveform with increasing voltage. (**B**) Representative slices post PFA treatment with 100, 300, and 500 V in the XY and Z plane. (**C**,**D**) Quantification of lesion characteristics including depth, volume, and area. Data are mean ± SEM. *n* ≥ 3. **** *p* < 0.0001, ns not significant.

To test the effect of varying voltage we used a pulse generator that delivered increasing amounts of voltage to slices connected to an oscilloscope that captures the waveform characteristic (Figure 2A). Representative images for 100, 300, and 500 V show lesion boundaries in the XY and Z dimensions (Figure 2B).

A significant increase in lesion size was observed for voltage ranges from 100 to 700 V and different parameters like depth, volume, and area are quantified (Figure 2C,D). We also performed lesion length and width measurements (Figure S2A). These results demonstrate that voltage had a large impact on circularity, and ST slices accurately capture voltage-dependent ablation outcomes, a key determinant of PFA treatment design.

### 2.3. Impact of Duration on Lesion Size

**Figure 3:**
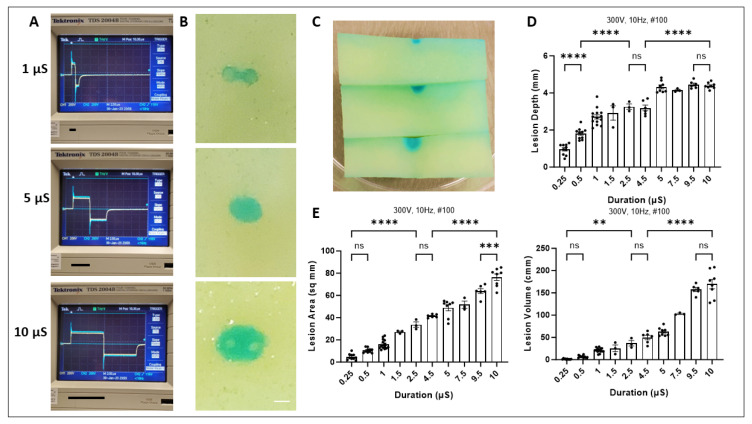
Effects of duration on ablation outcome. (**A**) Oscilloscope recordings with biphasic square waveform with increasing phase duration. Representative slices post PFA treatment with 1, 5, and 10 µS in XY and Z plane (**B**,**C**) Scale Bar 1 mm. Quantification of lesion characteristics including depth, volume, and area (**D**,**E**). Data are mean ± SEM. *n* ≥ 3. ** *p* < 0.01, *** *p* < 0.001, **** *p* < 0.0001, ns not significant.

Next, we tested the effects of increasing duration on ablation outcomes (Figure 3A). Representative slices post staining show changes in lesion shapes and sizes at 1, 5, and 10 µS (Figure 3B,C). The maximum depth and volumes quantified with increasing phase duration were significantly different at lower and higher ends of the spectrum. Subtle increments in steps of 0.5 µS may not necessarily affect lesion sizes in certain cases; however, larger microsecond step increases significantly increase lesion boundaries (Figure 3D,E). We also performed lesion length and width measurements with increasing duration (Figure S2B) and found similar increasing trends.

### 2.4. Modulating Frequency and Pulse Number Alter Lesion Size and Depth

**Figure 4:**
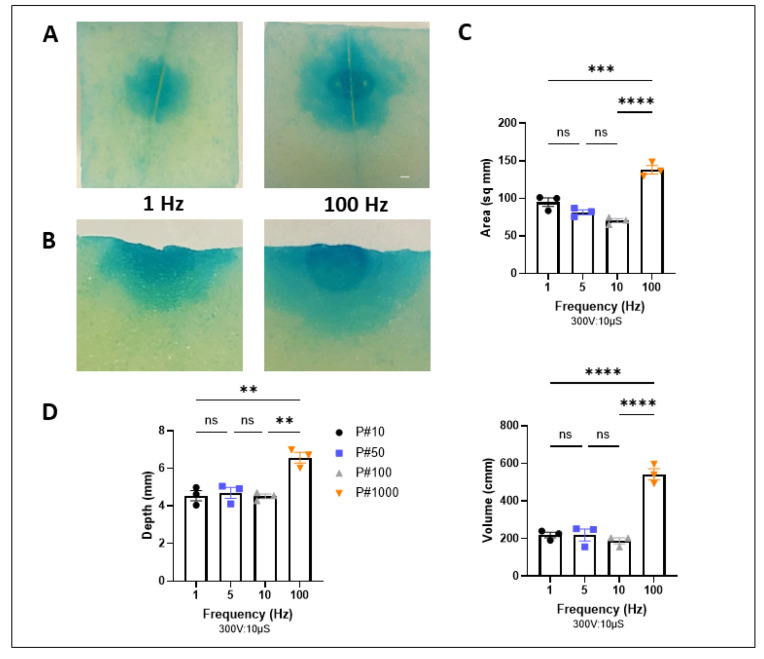
Frequency modulation with constant duration. (**A**) Representative slices post PFA treatment at 1 and 100 Hz in XY plane. Scale Bar 1 mm. (**B**) Slices post PFA treatment at 1 and 100 Hz in Z plane. Quantification of lesion characteristics including depth, volume, and area with increasing frequency and pulse number (**C**,**D**). Data are mean ± SEM. *n* ≥ 3. ** *p* < 0.01, *** *p* < 0.001, **** *p* < 0.0001, ns not significant.

Along with the above-tested parameters, we investigated the effect of increasing the frequency and pulse number on lesion size. With a constant total pulse duration, major changes in frequency showed increased lesion sizes (Figure 4A,B). Large increase in repetition rate from 1 to 100 Hz increased lesion boundaries, but subtle changes in frequency with constant total duration did not seem to significantly alter ablation outcome (Figure 4C,D and Figure S2D).

**Figure 5:**
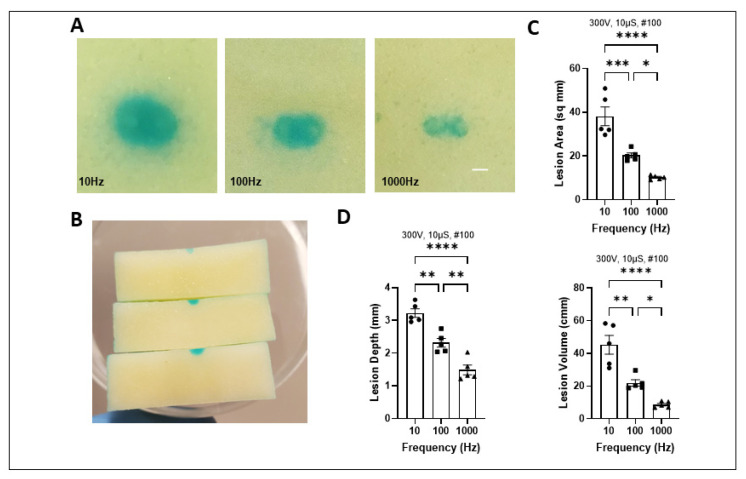
Frequency increase with total duration reduction. (**A**) Representative slices post PFA treatment at 10, 100, and 1000 Hz in XY plane. (**B**) Slices post PFA treatment at 10, 100, and 1000 Hz in Z plane. Quantification of lesion characteristics including depth, volume, and area with increasing frequency and decreasing total duration (**C**,**D**). Scale bar 1 mm. Data are mean ± SEM. *n* ≥ 3. * *p* < 0.05, ** *p* < 0.01, *** *p* < 0.001, **** *p* < 0.0001.

However, when frequency was increased with constant pulse number, thereby decreasing the total pulse duration, we observed a significant reduction in ablation outcomes. Representative slices (Figure 5A,B) highlight lesion characteristics with constant pulse number and increasing frequency in the XY and Z plane. The ablation outcome is quantified in Figure 5C,D and Figure S2C, highlighting size reduction trends.

### 2.5. Temperature Changes during PFA

**Figure 6:**
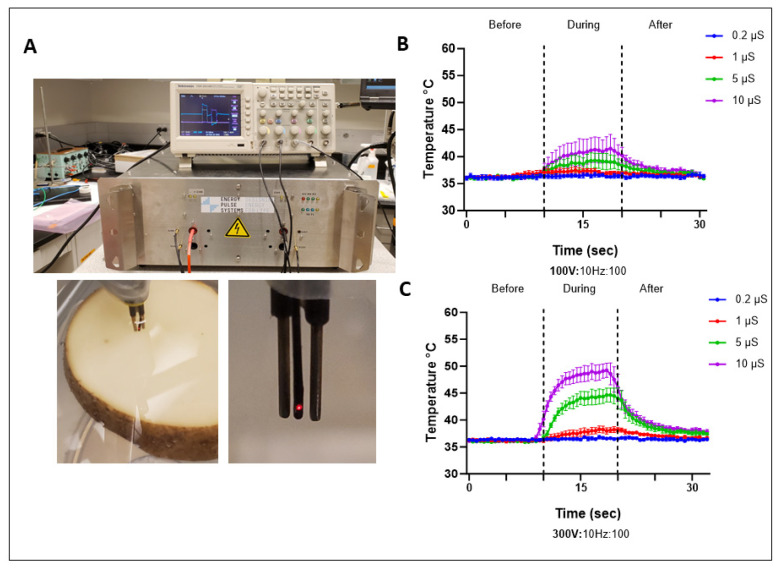
Temperature assessment with PFA. (**A**) Temperature assay setup with pulse generator, oscilloscope, and temperature probe at surface of slice between electrodes. (**B**,**C**) Temperature recordings before, during, and after PFA treatments with 100 and 300 V at different durations. Data are mean ± SEM. *n* ≥ 3.

We characterized changes in temperature using a fiber optic probe with a fast sampling rate. The probe was tested with different environments like ice water, room temperature air and water, water bath, and hot water to observe response dynamics and stabilization (Figure S3A,B). For PFA experiments, the probe was placed between the electrodes (Figure 6A), and temperature was recorded before, during, and after treatment. Before pulsing, the temperature was maintained at 37 °C, which gradually showed an increase with increasing voltage and duration, and at 10 µS with 300 V, the temperature reached ~48 °C (Figure 6B,C). We also measured temperature with the buffer alone and found similar trends at the higher voltage and durations tested (Figure S3C,D). Although the highest observed rise in temperature quickly returned to baseline within seconds after the treatment was terminated (Figure 6C), our results highlight the importance of measuring temperature data for the combination of treatment parameters for quantitative assessment of thermal effects.

### 2.6. Electric Field Simulation and Modeling

**Figure 7:**
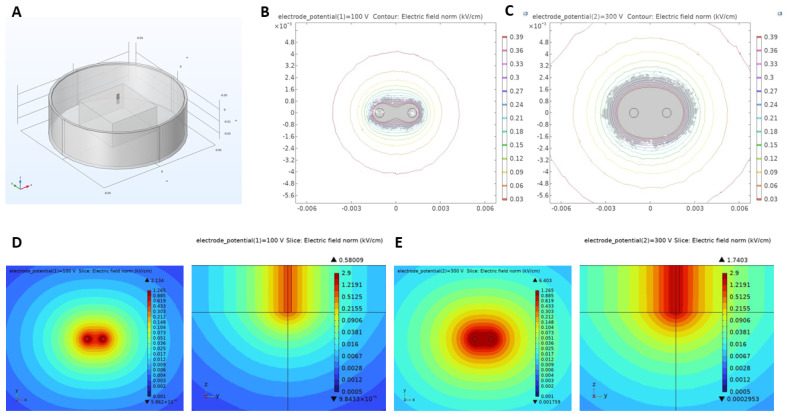
In silico modeling and EF distribution. (**A**) 3D illustration of finite-element simulation. (**B**,**C**) Stained slices of lesions obtained after PFA treatments of 100 and 500 V superimposed on electric field isolines. (**D**,**E**) Electric field distribution simulations in both XY and Z planes for PFA treatments of 100 and 300 V.

We ran numerical simulations to compute the electric field distribution that mimics the geometry, materials, and experimental setup. The ablation setup and electrode 3D geometry are illustrated in Figure 7A using COMSOL Multiphysics 5.6 software for numerical modeling. An example of stained lesion of vegetal slice after PFA treatment (100 V and 300 V) superimposed on contour lines generated via electric field distribution simulation (Figure 7B,C). The external edge of lesions corresponds to the minimum electric field necessary to induce the effect. We found that at 100 pulses of 10 uS the electric field threshold was 240 V/cm at a frequency of 10 Hz. Using in silico modelling, the electric field distribution is represented for pulses at 100 and 300 V (Figure 7D,E) in both the XY and Z plane, a key indicator for transmurality. Using the 3D tuber model allows for the demonstration of the Z plane distribution of the electric field which is beneficial as a different thickness across locations can affect penetration.

## 3. Discussion

This study focuses on the refinement of an in vitro assay platform that can be used in the early phase of device development to assess relationships across a range of clinically relevant pulse parameters and ablation outcomes. Data addressing the individual impact of pulse parameters on lesion volume and depth in a comprehensive, systematic, and controlled fashion are limited. Here, we aimed to address this potential gap, and the main findings include key determinants of IRE like duration, voltage, and frequency which have a strong correlation with lesion depth and volume. When accounting for frequency, the number of pulses plays a critical role in determining lesion size. When frequency increases with constant duration based on pulse number, an increase in lesion size is observed, whereas with increasing frequency with decreased total time there is a reduction in the ablation outcome characteristics. This highlights the importance of pulse number and application cycles that need to be validated in clinical regimen to achieve effectiveness during ablation. While a direct voltage increase significantly increases ablation volume, it may also augment thermal effects and contractility. Our data highlight that temperature changes were dependent on the specific combination of pulse parameters. To increase the power of bench testing, we supplemented in vitro assays with in silico modeling. Numerical simulations show the E-field distribution in both the XY and Z plane which can act as an indicator for transmurality and penetration depending on the upstream treatment regimen. Numerical models were designed to simulate the two-electrode placement on the tuber surface. A computational model compared the electroporated area between potatoes from the simulation and experiment to obtain electroporation threshold values of 240 V/cm. There is a wide range of electric field thresholds in tuber models ranging from 184 V/cm to 480 V/cm [25,26,27,28,29]. In a similar line, EFTs for other models like hiPSC-derived cardiomyocytes, ex vivo porcine, and human heart models show ranges from 363 V/cm to 1 kV/cm [20,30,31,32]. This wide range is not surprising as waveform parameters like pulse number, frequency and duration variability, tissue biophysical properties, and surrounding medium affect observed threshold values. A systematic analysis is warranted to compare PEF protocols to determine cellular differences in lethal EFTs and caution must be exercised as accuracy depends on multiple underlying factors like conductivity, pulse parameters, ablation setup, and segmentation. The observed values come close to the theoretical predictions obtained by means of numerical simulations. To further strengthen the qualitative outcomes of lesion characteristics we are working towards a quantitative assessment of the complex relation between independent pulse recipes and ablation outcomes using machine learning algorithms. Another factor to consider is the effect sizes that supplement the power analysis. For our ablation setup, higher pulse parameters show differences of biological interest and also ranges for minimum meaningful effects of scientific relevance. Fitting of bench data using prediction analysis can be used as a supplementary tool and can reduce animal burden in the early device development stage. To further extend this, our lab is working towards cross validating bench data with machine learning algorithms to predict ablation outcomes. This may also aid regulatory decision making with an emphasis on device efficacy and risk analysis.

### 3.1. Simplified Model

Over the last few years, there has been an emphasis on reducing animal burden (3R’s) and the development of new alternate methods to study effects of IRE. Vegetal model lesions can be allowed to develop overtime with oxidation by polyphenol oxidases or staining for acute lesion quantification. Currently there is no direct non-invasive lesion assessment in humans, and although this non-human model is rapid and noninvasive, certain limitations of this simplified model must be factored in while interpreting results. Electric field distribution can vary based on permittivity and conductivity which varies in living tissues both in plants and animals based on water and ionic content [17,33,34]. Another key difference that alters conductivity distribution in plant cells is the turgor pressure. The cell vacuoles maintain high pressure that mediate cell membrane and cell wall attachment. Changes in turgor pressure and osmotic forces can rearrange the structure and surrounding medium post electroporation, depending on the model used [35]. The stained lesion boundaries are relative markers for the electric field distribution and do not consider contractility effects on lesion penetration. We also acknowledge that although this model offers 3D lesion visualization, it does not account for human tissue architecture and cell-to-cell interactions in the cardiac environment.

### 3.2. Compare and Contrast with In Vivo or Cell Line Ablation Outcomes

Recent studies have also attempted to compare the geometry of electroporated areas using plant-based models with limited animal data. In a study by Lindelauf et al. [17], the authors studied a plant model that was compared to a retrospectively evaluated swine liver IRE dataset for similar conditions. The electroporated areas showed spherical geometry of comparable sizes and thus are speculated as promising quantitative predictors in animal tissue. Another study by Gasperetti et al. [27] used Yorkshire pigs to confirm findings and ablation effects from a similar potato model. These models for PFA assessment demonstrated lesion characteristics to be strongly dependent on voltage and number of cycles, with a quasi-linear relationship. A study by Sano et al. [9] used a potato and ex vivo liver model to compare ablations using a single electrode configured with a grounding pad vs. two collinear electrodes and demonstrated ablation zones that appeared to be of clinically relevant size and shape. Although rigorous qualitative and quantitative assessment is warranted, plant-based models can act as early-stage filtration tools to screen a myriad of upstream pulse parameters. This, in turn, can reduce animal burden particularly for overly aggressive scenarios post validation with in vivo and in silico approaches.

### 3.3. Future Impact

Several study reports tend to have fixed PFA protocols and pulse waveforms are often black boxes which make it difficult to assess key determinants of ablation outcomes. In this 3D vegetal model for PFA assessment, lesion depth was found to be strongly dependent on the pulse recipe. In the future, if operators are allowed to change procedural settings like pulse number, waveform, and frequency for a better treatment regimen catered for personalized medicine, it is essential to investigate ablation outcomes and determine safety thresholds correlating to upstream waveform modifications. Another factor that can alter the ablation outcome is contact force [36,37,38]. During PF ablation, there may be no detectable lesion formation without electrode contact, indicating the need for electrode tissue contact; however, more investigation is warranted to understand how significantly different force ranges modulate depth or lesion volume [39,40,41]. This model can act as a platform to assess accuracy and force measurements to determine range, catheter-to-tissue angle, and visualize lesion depth at high contact forces. Along with the pulse recipe, high-frequency protocols, catheter design, and electrode geometry can have an impact on ablation outcome [32,38,42]. Due to the option of variable slice sizes and thickness, this assay can be applied to custom electrodes with varying geometry with needle-shaped, spline, balloon, circular, and other large-footprint catheters. Another avenue for adoption of this model is the repurposing of other energy modality catheters like radiofrequency (RF) and cryoablation that may be used in future pulsed energy delivery methods. Testing of dual ablation modalities [40] is another avenue for future work, where a systematic screening of both energy delivery and its ablation outcome need a high-throughput and robust platform, for which this vegetal model can be applied as it is high-throughput, cost-effective, and lesions are visible rapidly. Although species variation for plant- and animal-based models are yet to be studied in-depth and will evolve with this newer ablation modality, refinement of bench assays is critical for long term model adoption. Personalized pulse recipe optimization may present as a tradeoff of effectiveness versus long-term stability and future studies are warranted that assess the chronic effects of PF ablation.

## 4. Materials and Methods

### 4.1. Sample Preparation

Russet potatoes (*Solanum tuberosum*) were obtained from a local market and stored at room temperature for no more than 7–10 days until used for experiments. PFA lesions were delivered on either square or circular vegetal specimens placed in a tray containing Tyrode buffer (140 mM NaCl, 5 mM KCl, 2 mM CaCl_2_, 2 mM MgCl_2_, 5 mM HEPES, 10 mM Glucose, and adjusted to a pH 7.2 using NaOH) using two circular electrodes with a dedicated IRE generator. The temperature was maintained at 37 degrees with a water bath. Different pulse parameters like voltage, duration, and frequency were tested in a randomized manner, and the tissue slice labels were blinded during both manual and automated image analysis. After an hour resting period, the samples were immersed in commercial blue coloring dye for another 30 min. Excess dye was rinsed with water and lesions were visualized in both the XY and Z planes. The potato slices were kept in covered petri dishes or multi-well plates at room temperature for a total of ~ 4 h. Photographs were taken under constant illumination with a 12 MP (1/2.55”) sensor with variable aperture f/1.5–2.4 and big 1.4 µm pixels using a Samsung (Ridgefield, NJ, USA) digital camera.

### 4.2. PF Ablation Setup

A pair of custom stainless-steel needle electrodes (0.63 mm diameter and 1.37 mm inter electrode distance) were connected to an electric pulse generator, the model pulse generator EPULSUS-FBM1-5 (EnergyPulse Systems, Lda., Lisbon, Portugal). Waveforms were measured with an oscilloscope (Tektronix, Beaverton, OR, USA) connected to a voltage probe, model P2501 (Owon Technology Inc., Zhangzhou, China). For a majority of the experiments, a continuous sequence of 100 biphasic, symmetrical, rectangular pulses with a 500 ns interphase delay were applied. Treatment parameters varied between samples and ranges include phase amplitude (100, 300, 500, 600, and 700 V), duration (0.25, 0.5, 1, 1.5, 2.5, 4.5, 5, 7.5, 9.5, and 10 µs), and frequency (1, 5, 10, 100, and 1000 Hz). The electrodes were positioned perpendicular over the vegetal slice, and contact was confirmed at the surface. The slice was placed in a tray with Tyrode solution and the temperature was maintained using a Lindberg-Blue water bath.

### 4.3. EFT Calculations and Field Distribution Modeling

The AC/DC module of the COMSOL Multiphysics software (COMSOL Inc., Stockholm, Sweden) was used for finite element modeling of the electrical field distribution in in vitro geometry. A cylindrical glass dish with a height of 3 cm and an inner diameter of 9.6 cm and thickness of 2 mm was created. The inside of the glass dish was filled with a cylinder with Tyrode media with an electrical conductivity of 1.8 S/m and relative permittivity of 1. A cubic potato slice with a geometry of 4 cm length and 2 cm height and an electrical conductivity of 0.04 S/m and relative permittivity of 1 was placed at the center and 4 mm inside the top boundary of the Tyrode media. Two electrodes with a diameter of 0.63 mm, length of 8 mm, electric conductivity of 1.74 mS/m, and relative permittivity of 1 were placed inside the Tyrode media, touching the upper boundary of the potato slice with an inner electrode boundary distance of 1.37 mm. One electrode was connected to a voltage of 100–300 V, and the other electrode was connected to 0 voltage (ground). The insulation boundary condition was used for the inner boundary of the glass dish and air, due to its very low conductivity. Electric current physics with a stationary simulation was used to calculate the electric field inside the Tyrode cylinder. An extremely fine element size was chosen for the physics-controlled mesh, and a convergence plot was used to confirm the convergency of the simulation.

### 4.4. Image Analysis

We developed a JavaScript application with an HTML front-end to calculate the area and the volume of the lesion from the JPG/PNG pictures of the lesions. The application receives a picture of the top view as well as a picture of the cut view of the lesion. We use separate brightness thresholds for the top-view and the cut-view images to mark the stained area which represents the lesion area. The tool has a built-in feature to calculate the physical distance between any two points on the images based on the overall size of the region that the image represents. This feature is used to manually select and measure the length and depth of the lesion from the top view and cut views, respectively. The area of the lesion is then calculated by first simply counting the number of pixels that are marked as stained which fall below the prescribed brightness threshold and subsequently multiplying the number of pixels by the area of the pixel. The volume calculations are more involved and require a key assumption about the magnitude of the electric field in the third dimension. We assume that the distance between the two electrodes is small compared to the volume of interest. Subsequently, our numerical modeling shows that the equipotential lines of the electric field are geometrically similar between the top view and the cut planes that are parallel to the top-view plane. Under such conditions, all corresponding lengths of two similar shapes have the same ratio, and, subsequently, the area of the two shapes is proportional to the square of that ratio. Therefore, assuming that A(z) represents the area of the lesion on a plane that is parallel to the top view and z is the distance from the top view, the volume of the lesion is $V=\int_{0}^{d}A\left(z\right)dz$ where V is the volume of the lesion and d is the depth of the lesion. We calculate this integration using the trapezoidal integration rule [43]. To be able to estimate $A\left(z\right),$ we need a reference line on the top surface which corresponds to the cutting line on the cut-view image. The user manually selects this line in the developed application, on the top-view image. On the cut-view image, the lesion area is marked by a polygon when the user selects the two points that correspond to the same line in the top view. Then, the user progressively chooses the points on the curved boundary of the lesion to closely mark it. In the end, this process marks the lesion area as a polygon. The perpendicular bisector of the line corresponding to the top cut line is used to represent z (depth axis). The curve boundary is then used to automatically find the area of the lesion on the plane at the depth z by calculating the ratio of the distance between the cross section of the curved boundary and the plane at depth z and the length of the top line. A(z) will be the square of that ratio multiplied by the area of the lesion on the top view. This process is repeated for all $A\left(z\right)$ s that are used for the trapezoidal rule volume calculation. The calculated volume is subsequently displayed in the application.

### 4.5. Temperature Measurements

Temperature changes resulting from PFA treatments were monitored using a non-metallic optic STB probe (catalog # L-00-14500-01, Advanced Energy Industries, Denver, CO, USA) with a response time of 0.25 s, sampling rate of 0.02 s, and diameter of 0.5 mm. The STB probe was positioned adjacent and parallel to one of the electrodes. The probe’s performance was evaluated both with and without slices and was exposed to various temperatures, as detailed in Figure S3. Temperature measurements were taken before, during, and after treatments to capture the complete temperature profile, including the rate of increase and decrease in temperature.

### 4.6. Statistical Analysis

Prism 8 software (Prism 8, GraphPad Software, Boston, Massachusetts USA) was used for statistical analysis. Differences among the groups are presented as mean ± standard error of the mean (SEM). Differences were assessed as compared with lesion measurements using the standard one-way ANOVA and hypothetical values of zero. Multiple comparisons used follow-up tests to compare the mean of each column with the mean of every other column. Correction for multiple comparisons was performed with Tukey’s test. * *p* < 0.05, ** *p* < 0.01, *** *p* < 0.001, **** *p* < 0.0001, and ns not significant. Results were considered statistically significant if the *p*-value was less than 0.05.

## 5. Conclusions

In this study, we have developed a robust, rapid, and scalable assay protocol for efficient testing of a wide range of PFA parameters in a 3D vegetal model for ablation. This work lays the foundation for a standardized nonclinical bench testing assay that can facilitate the characterization of PFA waveforms and ablation effects, support device development, and aid in regulatory decision making. Key findings from this study include the following: (i) refinement of an in vitro assay, enabling high-throughput, noninvasive visualization of lesion boundaries following PFA treatment; (ii) identification of pulse parameter variations, particularly in duration, voltage, and frequency, that modulate ablation outcomes in terms of 2D (length, width, and area) and 3D (depth and volume) effects; (iii) development of an alternative method to test the combination of pulse parameters to asses temperature changes before, during, and after PFA treatments; and (iv) cross-validation of experimental data with in silico modeling allows for the assessment of the E-field distribution and lethal thresholds for a wide array of clinically relevant pulse recipes. These findings highlight the potential of our assay development for advancing PFA research and application, offering a valuable tool for researchers and device developers in the field.

## Supplementary Materials

The following supporting information can be downloaded at: https://www.mdpi.com/article/10.3390/ijms25168967/s1.
