# Supplementary material for: In Vitro Assay Development to Study Pulse Field Ablation Outcome Using Solanum Tuberosum
Source: Int J Mol Sci. 2024 Aug 17;25(16):8967. doi: 10.3390/ijms25168967 (PMC11354718; doi:10.3390/ijms25168967)
Supplement: Supplementary file 1 [file ijms-25-08967-s001.zip › ijms-3121377-supplementary.pdf]

Supplementary Figures:

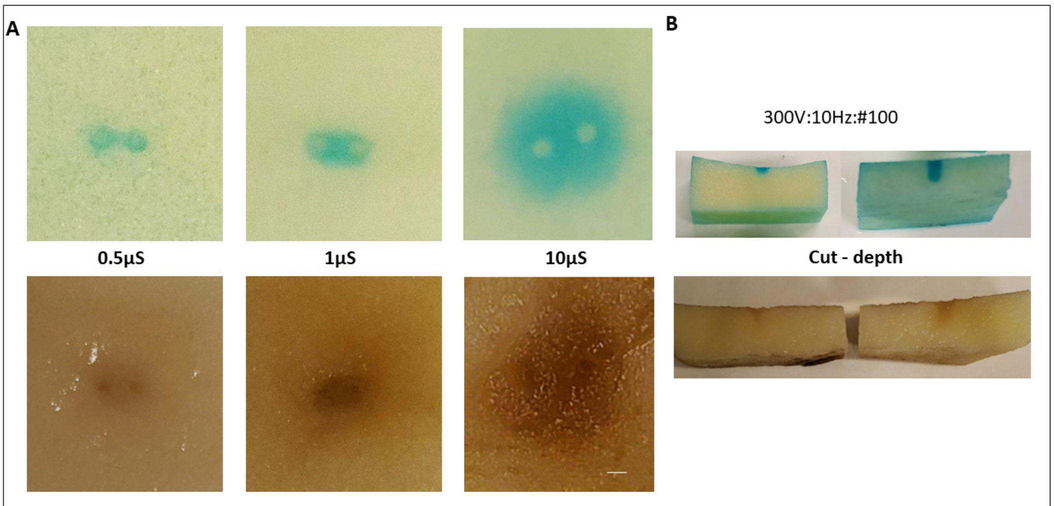

Figure S1.

Figure S1: Lesion boundary staining assay.

A. Representative XY lesions stained (top row) and no dye (bottom row) for increasing durations post PFA treatments. B. Similar Z sections depicting depth for stained and unstained slices.

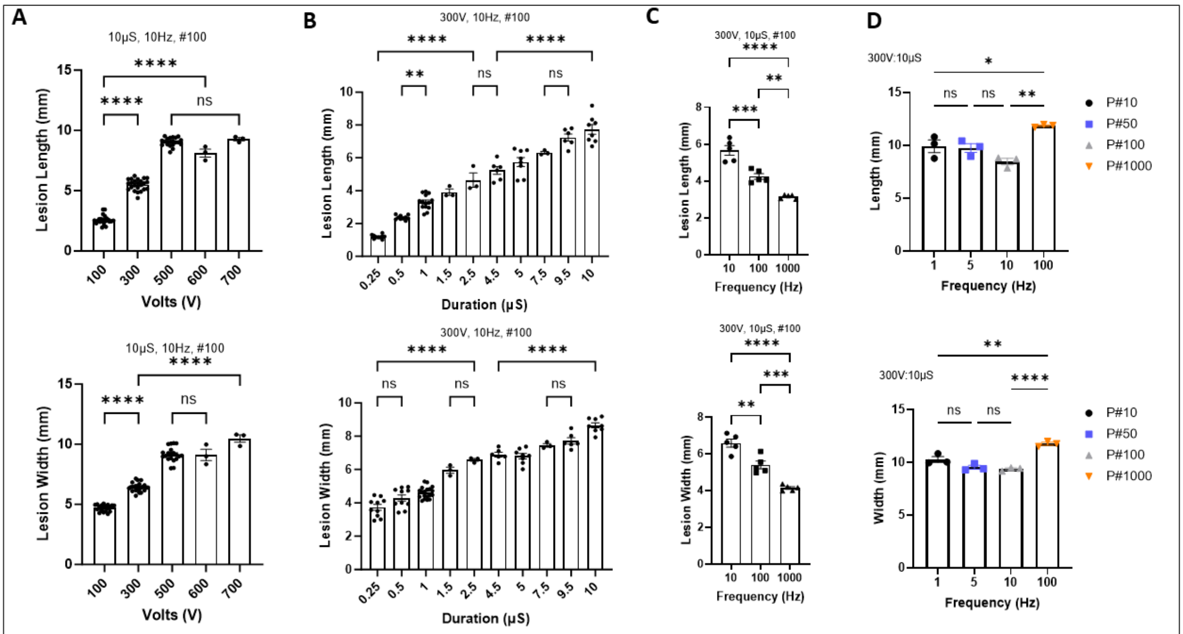

Figure S2.

Figure S2: Lesion measurements

A. Quantification of lesion length and width for increasing voltage. B. Quantification of lesion length and width for increasing duration. Analysis of lesion length and width for increasing frequency with constant pulse number and similar quantification for increasing frequency with variable pulse number (C, D). Data are mean ±SEM. n ≥ 3. *p < 0.05, **p < 0.01, ***p < 0.001, ****p < 0.0001, n.s. not significant

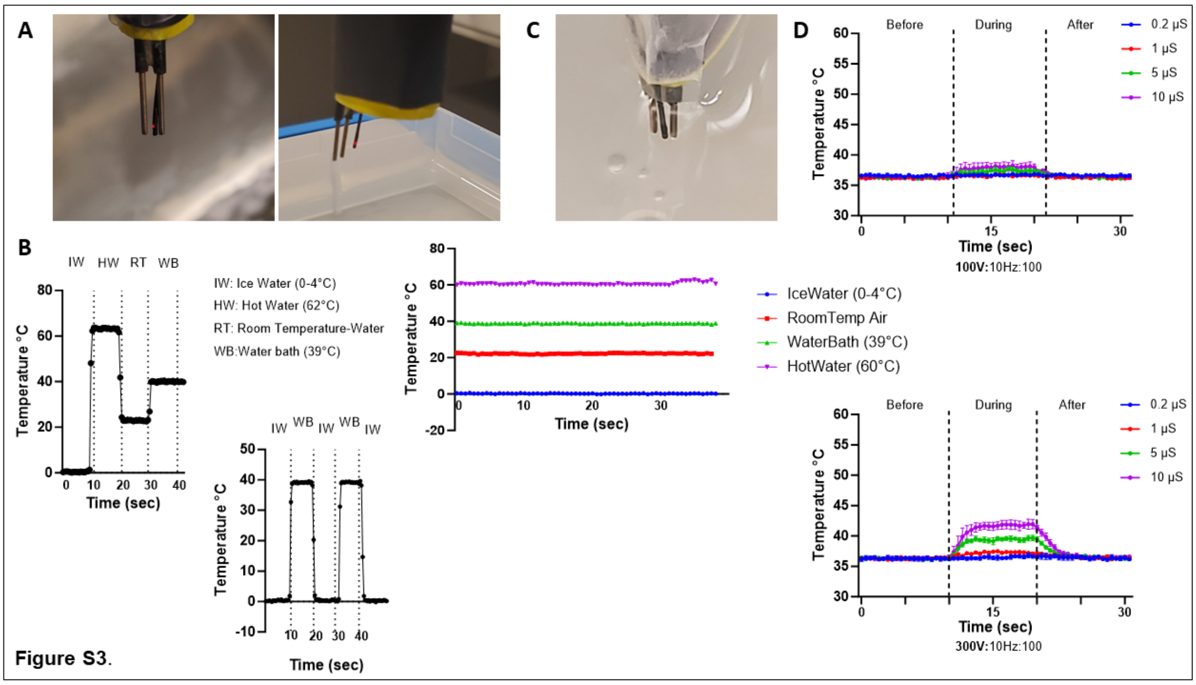

Figure S3: Temperature probe characterization

A. Images show probe placement between and outside the two electrodes. B. temperature readings taken over several seconds post exposure to different environments. C. Probe placed in Tyrode solution alone and temperature recordings before, during and after stimulation.
